# Alkyl chain length of quaternized SBA-15 and solution conditions determine hydrophobic and electrostatic interactions for carbamazepine adsorption

**DOI:** 10.1038/s41598-023-32108-3

**Published:** 2023-03-30

**Authors:** Jin-Kyu Kang, Hyebin Lee, Song-Bae Kim, Hyokwan Bae

**Affiliations:** 1 Institute for Environment and Energy, Pusan National University, 2 Busandaehak-ro 63beon-gil, Geumjeong-gu, Busan, 46241 Republic of Korea; 2 Graduate School of Carbon Neutrality, Ulsan National Institute of Science and Technology (UNIST), 50 UNIST-gil, Eonyang-eup, Ulju-gun, Ulsan, 44919 Republic of Korea; 3 Environmental Functional Materials and Water Treatment Laboratory, Department of Rural Systems Engineering, Seoul National University, 1 Kwanak-ro, Kwanak-gu, Seoul, 08826 Republic of Korea; 4 Department of Urban and Environmental Engineering, Ulsan National Institute of Science and Technology (UNIST), 50 UNIST-gil, Eonyang-eup, Ulju-gun, Ulsan, 44919 Republic of Korea

**Keywords:** Pollution remediation, Environmental monitoring

## Abstract

Santa Barbara Amorphous-15 (SBA) is a stable and mesoporous silica material. Quaternized SBA-15 with alkyl chains (Q_SBA_) exhibits electrostatic attraction for anionic molecules via the N^+^ moiety of the ammonium group, whereas its alkyl chain length determines its hydrophobic interactions. In this study, Q_SBA_ with different alkyl chain lengths were synthesized using the trimethyl, dimethyloctyl, and dimethyoctadecyl groups (C1Q_SBA_, C8Q_SBA_, and C18Q_SBA_, respectively). Carbamazepine (CBZ) is a widely prescribed pharmaceutical compound, but is difficult to remove using conventional water treatments. The CBZ adsorption characteristics of Q_SBA_ were examined to determine its adsorption mechanism by changing the alkyl chain length and solution conditions (pH and ionic strength). A longer alkyl chain resulted in slower adsorption (up to 120 min), while the amount of CBZ adsorbed was higher for longer alkyl chains per unit mass of Q_SBA_ at equilibrium. The maximum adsorption capacities of C1Q_SBA_, C8Q_SBA_, and C18Q_SBA_, were 3.14, 6.56, and 24.5 mg/g, respectively, as obtained using the Langmuir model. For the tested initial CBZ concentrations (2–100 mg/L), the adsorption capacity increased with increasing alkyl chain length. Because CBZ does not dissociate readily (pK_a_ = 13.9), stable hydrophobic adsorption was observed despite the changes in pH (0.41–0.92, 1.70–2.24, and 7.56–9.10 mg/g for C1Q_SBA_, C8Q_SBA_, and C18Q_SBA_, respectively); the exception was pH 2. Increasing the ionic strength from 0.1 to 100 mM enhanced the adsorption capacity of C18Q_SBA_ from 9.27 ± 0.42 to 14.94 ± 0.17 mg/g because the hydrophobic interactions were increased while the electrostatic attraction of the N^+^ was reduced. Thus, the ionic strength was a stronger control factor determining hydrophobic adsorption of CBZ than the solution pH. Based on the changes in hydrophobicity, which depends on the alkyl chain length, it was possible to enhance CBZ adsorption and investigate the adsorption mechanism in detail. Thus, this study aids the development of adsorbents suitable for pharmaceuticals with controlling molecular structure of QSBA and solution conditions.

## Introduction

The ever-increasing production, consumption, and release into environment of pharmaceutical and personal care products (PPCPs) has become a global concern^1,2^. Carbamazepine (CBZ) is one of the four most widely prescribed pharmaceuticals for the treatment of epilepsy and psychosis^3,4^. The extensive use and long durability/low degradability of CBZ have resulted in its detection in sewage, surface water, groundwater, and drinking water^4,5^. CBZ cannot be treated adequately using conventional water treatments. Therefore, research efforts are underway to remove CBZ from the aqueous phase using advanced methods, such as filtration^6–8^, biological processes^9^, advanced oxidation methods^5,8–12^, coagulation/flocculation/sedimentation^13,14^, and adsorption^3,14–20^. Among the various methods being explored, adsorption is particularly attractive because it is simple in design, easy to perform, cost-effective, and free of byproducts^15,16^.

Since carbon-based materials (CBMs) have high specific surface areas and hydrophobic characteristics, they are being explored for use in various fields^21–23^. They have also been studied widely for use as adsorbents with high sorption capacities for organic compounds^24–28^. Zhu et al.^29^ reported that the octanol/water distribution coefficient with respect to dissociation at pH 7 is proportional to the adsorption performance of the porous adsorbent or CBM used. They suggested that hydrophobic or π–π interactions are the major mechanisms of PPCP adsorption. Thus, the hydrophobic interactions between the PPCP in question and the adsorbent used have a determining effect on the adsorption process. The pK_a_ of an organic molecule determines the specific pH at which protonation or deprotonation occurs. Hence, PPCPs deprotonate and form negative ions at pH < pK_a_, which inhibits the hydrophobic interactions between the CBM used and the PPCP^30,31^. In addition, the ionic strength of the aqueous phase also affects the hydrophilic and hydrophobic interactions^32^. Therefore, the adsorption efficiency of CBZ, which is a representative persistent PPCP and does not readily undergo biological and physicochemical degradation^33,34^, can be controlled based on the hydrophobicity of the adsorbent used and the environmental conditions such as pH and ionic strength.

Santa Barbara Amorphous-15 (SBA) is a stable and mesoporous silica material. The effects of the length of the alkyl chain attached to quaternized SBA-15 (Q_SBA_) on its hydrophobic and hydrophilic adsorption properties have been studied^35,36^. For example, Q_SBA_ with a long alkyl chain shows high adsorption for diclofenac (DCF) owing to both the hydrophobic interactions of the long alkyl chain and the electrostatic attraction of the N^+^ species^36^. For the nitrate ion, electrostatic adsorption on the N^+^ species of the quaternary ammonium occurs readily even in the presence of competing oxyanions, such as bicarbonate, phosphate, and sulfate ions. This is owing to the high nitrate selectivity of Q_SBA_ because of its long-alkyl-chain-based hydrophobicity from higher hydration energy of nitrate than one of the other oxyanions^35^. By controlling the hydrophobicity based on the length of the alkyl chain, the adsorption capacity and selectivity for the target contaminants can be improved, and the adsorption mechanism can be elucidated^35,36^. However, in previous studies, the adsorption characteristics have been investigated only with respect to PPCPs based on different functional groups^37,38^, and there has been no research on the adsorption characteristics of Q_SBA_ for PPCPs based on its hydrophobicity; the exception is DCF^36^. CBZ has a lower hydrophobicity than DCF but does not dissociate under general pH conditions because of its high pK_a_^39–42^. Therefore, its adsorption on Q_SBA_ is expected to be different from that of DCF in terms of the adsorption capacity, which would depend on the alkyl chain length. The results obtained for CBZ can be utilized to propose an appropriate alkyl chain length for Q_SBA_ in removing various PPCPs, considering their characteristics.

In this study, we examined the effects of the hydrophobicity of Q_SBA_ on CBZ adsorption by varying the alkyl chain length of Q_SBA_ as well as the aqueous conditions via batch experiments. The equilibrium adsorption capacity was evaluated by testing the effects of the initial CBZ concentration on its adsorption on Q_SBA_ with alkyl chains of different lengths (trimethyl, dimethyloctyl, and dimethyoctadecyl, which resulted in C1Q_SBA_, C8Q_SBA_, and C18Q_SBA_, respectively). It was hypothesized that the changes in the hydrophobicity of Q_SBA_ would result in variations in the kinetics and equilibrium adsorption characteristics for hydrophobic CBZ. The pH and ionic strength of the test solution were also varied. This study provides additional insights into the molecular structure of Q_SBA_ for CBZ adsorption as well as the optimal characteristics of wastewater for the removal of CBZ.

## Materials and methods

### Quaternized SBA-15

C1Q_SBA_, C8Q_SBA_, and C18Q_SBA_ were prepared using a previously reported method^35^. Briefly, C1Q_SBA_ was prepared according to the following procedure: 0.1 mol of trimethyl[3-(trimethoxysilyl)propyl]ammonium chloride (50% in methanol, Tokyo Chemical Industry, Tokyo, Japan) and 6 g of SBA (Sigma Aldrich, MO, USA) were stirred for 1 h in 100 mL of toluene (99.5%, Daejung, Siheung, Republic of Korea). The mixture was then refluxed with 1 mL of deionized (DI) water at 100 °C for 48 h. Next, the slurry was treated with 0.1 M NaCl, separated using a 0.45-μm polyvinylidene fluoride (PVDF) filter, and dried at 65 °C in a drying oven until use. C8Q_SBA_ and C18Q_SBA_ were also prepared using the same processes as that employed for C1Q_SBA_; the difference was that dimethyloctyl[3-(trimethoxysilyl)propyl]ammonium chloride and dimethyloctadecyl[3-(trimethoxysilyl)propyl]ammonium chloride (42% in methanol, Sigma Aldrich, MO, USA), respectively, were used instead of trimethyl[3-(trimethoxysilyl)propyl]ammonium chloride. Dimethyloctyl [3-(trimethoxysilyl)propyl]ammonium chloride was synthesized by reacting 0.1 mol of (3-chloropropyl)trimethoxysilane (≥ 97%, Sigma Aldrich, MO, USA) and 0.1 mol of N,N-dimethyloctylamine (95%, Sigma Aldrich, MO, USA) at 85 °C for 48 h.

### Test solution of CBZ

A 100 mg/L CBZ stock solution was prepared by dissolving 50 mg of CBZ (≥ 98%, Sigma Aldrich) in 10 mL of methanol and then diluting it to 500 mL with DI water. There was no shift in the λ_max_ value (285 nm) of CBZ with the pH (Fig. S1). Therefore, the calibration curves of CBZ were obtained by measuring the absorbance of a serially diluted solution at 285 nm using an ultraviolet–visible spectrophotometer (Optizen POP, Mecasys, Korea) and 1-cm quart cells for each pH.

### Batch experiments

Because CBZ has high hydrophobicity and low solubility in water, it is difficult to test a wide range of concentrations in aqueous solutions. Furthermore, the dissociation of molecules greatly influences their solubility. Therefore, in this study, we examined the solubility to determine the concentration range for dissociation based on Eq. (1)^39^:

1 $${S}_{H}={S}_{0}\left(1+\frac{{K}_{a}}{\left[{H}^{+}\right]}\right) $$

where S_H_ is the solubility in water at a specific pH (mg/L), and S_0_ is the intrinsic solubility of an undissociated molecule. Because the pK_a_ of CBZ is very high (13.9), CBZ remains intact with low solubility at pH < 13.9. Therefore, the batch experiments to confirm its adsorption were conducted at concentrations of 100 mg/L or less, as S_0_ is in the range of 112–236 mg/L.

Batch experiments were also performed for Q_SBA_ by varying the contact time, initial CBZ concentration, pH, and ionic strength. All these experiments were performed using 0.03 g of either C1Q_SBA_, C8Q_SBA_, or C18Q_SBA_. A 30 mL of the CBZ solution was poured into a 50-mL conical tube and incubated at 150 rpm and 25 °C in a shaking incubator. For each batch condition, the experiments were conducted in duplicate. After the reaction, the Q_SBA_ and solution were separated using a 0.45-μm PVDF filter. The CBZ concentrations before and after the batch experiment were calculated using a calibration curve.

The reaction times, CBZ concentrations, pH, and ionic strengths are listed in Table S1, which also lists the reaction conditions. The reaction time was varied from 5 to 360 min at a fixed CBZ concentration of 40 mg/L. In the other experiments, the CBZ solution and Q_SBA_ were allowed to react for 24 h. The equilibrium adsorption capacity was measured for various initial concentrations of CBZ (2–100 mg/L). The effect of the pH was assessed by adjusting the initial pH of the CBZ solution (40 mg/L) to 2, 4, 6, 8, and 10 using 0.1 M HCl and 0.1 M NaOH. The effect of the ionic strength was evaluated by adding 0.1–100 mM NaCl to the CBZ solution (40 mg/L).

### Data analysis

The amount of CBZ adsorbed during the reaction time (*q*_*t*_) experiments was determined by fitting the data using various kinetic models (pseudo-first-order^43^, pseudo-second-order^44^, and Elovich^45^ models, Table S1), while the amount of CBZ adsorbed at equilibrium (*q*_*e*_) during the initial concentration experiments was fitted using the Freundlich^46^, Langmuir^47^, and Redlich–Peterson^48^ models (Table S2). The optimal parameters for each model for each Q_SBA_ were obtained via nonlinear regression using the solver function in Excel 2019 (Microsoft Corporation, WA, USA). The coefficient of determination (Eq. 2) and sum of the squared error (Eq. 3) were used as the error functions for model comparison.

2 $${R}^{2}= \frac{{\sum }_{i=1}^{m}{{\left({y}_{c}-\overline{{y }_{e}}\right)}_{i}}^{2}}{{\sum }_{i=1}^{m}{{\left({y}_{c}-\overline{{y }_{e}}\right)}_{i}}^{2}+{\sum }_{i=1}^{m}{{\left({y}_{c}-{y}_{e}\right)}_{i}}^{2}}$$

3 $$SSE = {\sum }_{i=1}^{n}{{\left({y}_{e}-{y}_{c}\right)}_{i}}^{2}$$

where *R*^2^, Coefficient of determination; *SSE,* Sum of the squared error; *y*_*c*_, Adsorption capacity calculated using the model; *y*_*e*_*,* Adsorption capacity measured experimentally; $\overline{{y }_{e}}$, Average measured adsorption capacity.

### Characteristics of quaternized SBA-15 and CBZ

**Figure 1:**
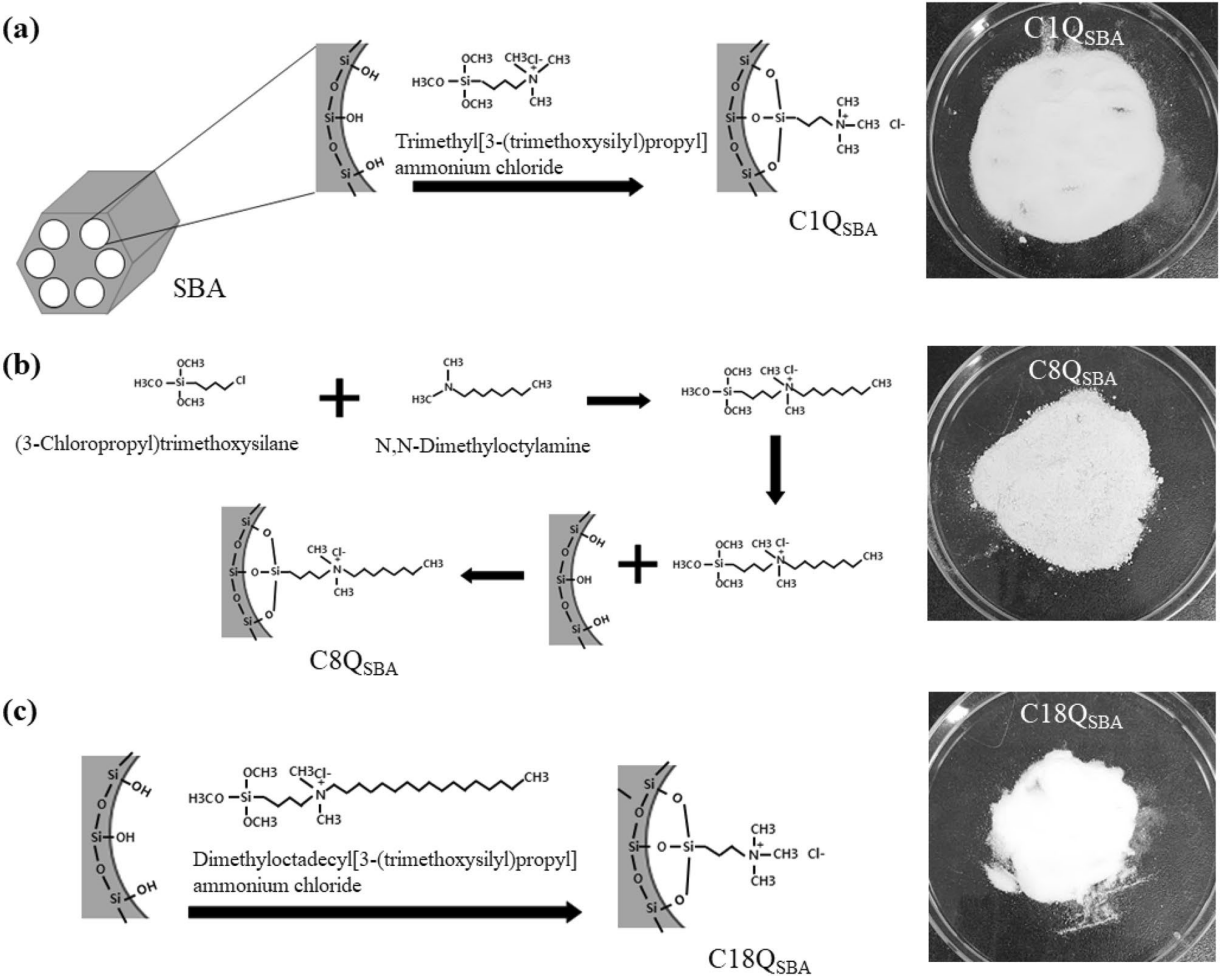
Schematics of the procedures for preparing Q_SBA_ samples and their digital images: (**a**) C1Q_SBA_, (**b**) C8Q_SBA_, and (**c**) C18Q_SBA_. Figure was modified from Kang and Kim^35^.

Figure 1 shows a schematic of the procedure for preparing the Q_SBA_ samples and digital images of the samples. X-ray photoelectron spectroscopy (XPS), ^13^C solid-state nuclear magnetic resonance (NMR) spectroscopy, and Fourier-transform infrared (FT-IR) spectroscopy were performed on C1Q_SBA_, C8Q_SBA_, and C18Q_SBA_ in a previous study^35^. XPS confirmed that quaternary ammonium was well-crosslinked to the SBA surface (Fig. S2). The ^13^C NMR and FT-IR spectra also showed that the alkyl chains were well grafted, as intended (Figs. S3 and S4, respectively).

Table 1 lists the major chemical characteristics of CBZ. The octanol–water partitioning coefficient (log K_ow_) value of 2.25 indicates that CBZ is hydrophobic. The high pK_a_ (13.9) indicates that, in aqueous solutions, CBZ exists in an undissociated state at almost all pH.

**Table 1:** Characteristics of CBZ.

Name	Chemical structure	Molecular weight	log K_ow_	pK_a_	Intrinsic solubility (S_0_)	Wavelength for measurement
Carbamazepine (CBZ)		236.27	2.45^40^	13.9^41^	112–236 mg/L^19^	285 nm^49^

## Results and discussion

### Adsorption kinetics

**Figure 2:**
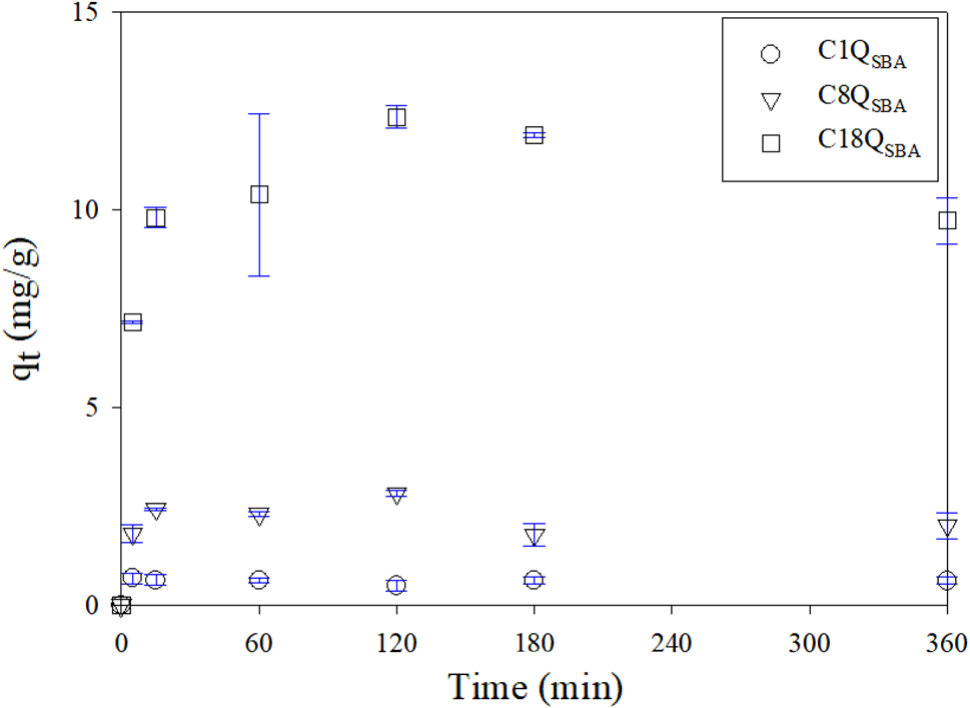
Effect of contact time on adsorption of CBZ by Q_SBA_.

Fig. 2 shows the temporal trend for the adsorption of CBZ by Q_SBA_. The adsorption process reached equilibrium within 120 min. Table 2 lists the parameters for the various kinetics models. The fitting quality as determined based on the coefficient of determination (R^2^) was the best in the case of the pseudo-first-order model (Fig. S5). The equilibrium q_t_ (i.e., q_e_) values for C1Q_SBA_, C8Q_SBA_, and C18Q_SBA_ as calculated using the pseudo-first-order model were 0.618, 2.279, and 10.988 mg/g, respectively. Thus, the q_t_ value varied with the alkyl chain length of Q_SBA_. CBZ adsorption was enhanced by increasing the alkyl chain length of Q_SBA_. In a previous study, C8Q_SBA_ showed an adsorption capacity as high as 593 mg/g for hydrophobic and dissociated DCF^36^. In this study, the hydrophobic adsorption of CBZ by C18Q_SBA_ was even greater, owing to the longer alkyl chain of the latter. Thus, it was confirmed that the adsorption of undissociated PPCP molecules can be significantly enhanced by using Q_SBA_ with a long alkyl chain.

**Table 2:** Kinetics model parameters for adsorption of CBZ by Q_SBA_.

Q_SBA_	Kinetics models											
	Pseudo-first-order				Pseudo-second-order				Elovich			
	q_e_ (mg/g)	k_1_ (1/min)	SSE (mg^2^/g^2^)	R^2^ (−)	q_e_ (mg/g)	k_2_ (g/mg/min)	SSE (mg^2^/g^2^)	R^2^ (−)	α (mg/g/min)	β (g/mg)	SSE (mg^2^/g^2^)	R^2^ (−)
C1Q_SBA_	0.618	3.623	0.0203	0.942	0.618	2440	0.0203	0.942	2.29 × 10^59^	236	0.0227	0.935
C8Q_SBA_	2.279	0.327	0.648	0.870	2.199	113	0.821	0.835	1.00 × 10^24^	28.5	0.817	0.836
C18Q_SBA_	10.988	0.198	5.051	0.953	11.4	0.0316	4.72	0.956	3.77 × 10^3^	1.22	8.46	0.921

The k_1_ value was calculated based on the fitted pseudo-first-order model to characterize the adsorption of CBZ by Q_SBA_. The k_1_ value decreased with increasing alkyl chain length, with the values for C1Q_SBA_, C8Q_SBA_, and C18Q_SBA_, being 3.623, 0.327, and 0.198 L/min, respectively. The trends for q_e_ and k_1_ were opposite because an increase in the alkyl chain of Q_SBA_ meant more adsorption sites and thus more time required to reach equilibrium.

### Effect of initial CBZ concentration

**Figure 3:**
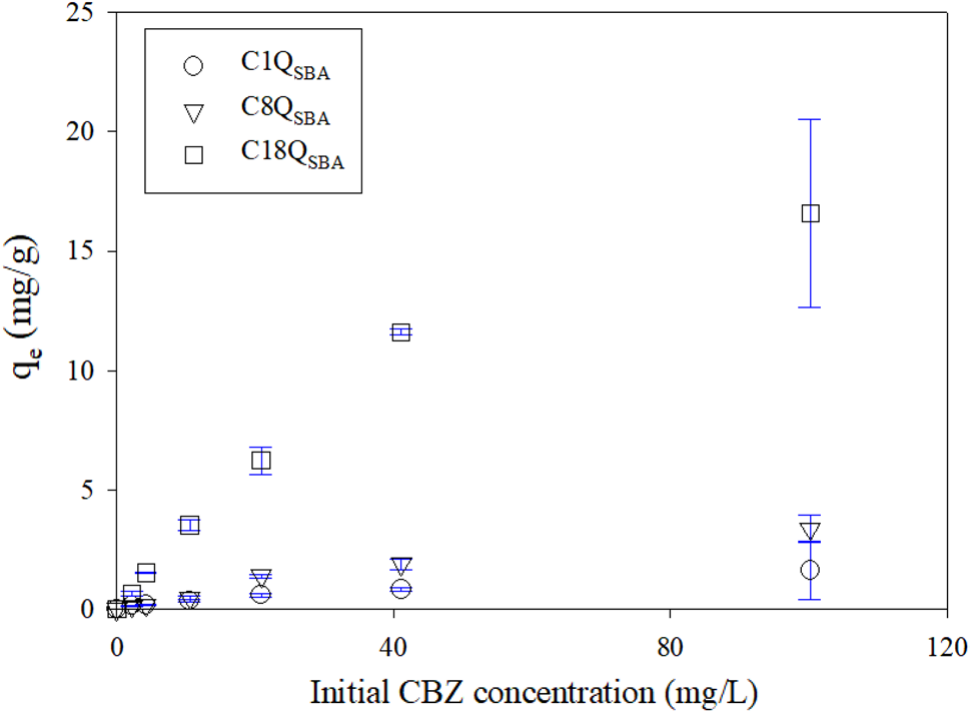
Effect of initial concentration of CBZ on its adsorption by Q_SBA_.

Figure 3 shows the effect of the initial concentration of CBZ on its adsorption by Q_SBA_. The observed data were analyzed using various isotherm models such as the Freundlich, Langmuir, and Redlich–Peterson models (Fig. S6). Table 3 lists the parameters of the isotherm models. Similar to the trend seen in the kinetics, the Q_SBA_ samples with longer alkyl chains exhibited higher maximum adsorption capacities (Q_m_) in the case of the Langmuir model (3.14, 6.56, and 24.5 mg/g for C1Q_SBA_, C8Q_SBA_, and C18Q_SBA_, respectively). As mentioned previously, the adsorption capacities of C8Q_SBA_ and C18Q_SBA_ were higher because of favorable hydrophobic interactions.

**Table 3:** Equilibrium model parameters for CBZ adsorption by Q_SBA_.

Q_SBA_	Equilibrium models												
	Freundlich				Langmuir				Redlich–Peterson				
	K_F_ (L/g)	1/n (−)	SSE (mg^2^/g^2^)	R^2^ (−)	Q_m_ (mg/g)	K_L_ (L/mg)	SSE (mg^2^/g^2^)	R^2^ (−)	K_R_ (L/g)	a_R_ (1/mg)	g (−)	SSE (mg^2^/g^2^)	R^2^ (−)
C1Q_SBA_	0.0734	0.672	0.00461	0.997	3.14	0.0104	0.0240	0.984	12.6	170	0.331	0.00463	0.997
C8Q_SBA_	0.135	0.709	0.184	0.977	6.56	0.0110	0.104	0.987	0.0720	0.0110	1.00	0.104	0.987
C18Q_SBA_	1.33	0.580	7.23	0.963	24.5	0.0263	1.36	0.993	0.644	0.0263	1.00	1.36	0.993

Regardless of the length of the alkyl chain, the Redlich–Peterson model was the most suitable of the three isotherm models used, based on their SSE and R^2^ values (Table 3). All three models showed high R^2^ values (> 0.967), which were acceptable for model fitting. This was probably because the experiments were not performed using high CBZ concentrations. The Langmuir model assumes monolayer adsorption, while the Freundlich model assumes multilayer adsorption^32^. However, both the Freundlich and Langmuir models showed linear adsorption characteristics within a certain early concentration range. Detailed characterization of the adsorption models, such as considering single and multiple layers, is not possible owing to the limited solubility of CBZ. Thus, the standard Langmuir model is a suitable one because Q_SBA_ would have a larger surface available for adsorption compared with that of CBZ. The adsorption characteristics of a number of PPCPs with limited solubility have been described previously using the Langmuir model, including CBZ^50–52^, ibuprofen^29^, levofloxacin^53^, sulfamethoxazole^54–56^, tylosin^55^, and 17β-estradiol^56^.

Table S3 compares the adsorption capacities of the various adsorbents for CBZ. Most adsorbents are based on activated carbon, mesoporous silica, or metal–organic frameworks. The data in Table S3 suggest that the primary mechanism for the adsorption of CBZ is hydrophobic interactions^16–20^. Deng et al.^19^ and Jun et al.^20^ reported Q_m_ values of 104.17 and 250.4 mg/g for CBZ using carbon-dot-modified magnetic carbon nanotubes and a metal–organic framework (Basolite A100), respectively. These values are much larger than that of C18Q_SBA_ (24.5 mg/g). However, no study has attempted to control the hydrophobicity of the adsorbent to confirm that hydrophobic interactions are indeed the adsorption mechanism responsible for the removal of CBZ. In this study, we show clearly that the removal of CBZ is improved owing to the higher hydrophobicity owing to the longer alkyl chains. We also analyzed the effects of the pH and ionic strength to confirm that the enhancement in the hydrophobic interactions is not affected by the various wastewater characteristics.

### Effect of initial pH

**Figure 4:**
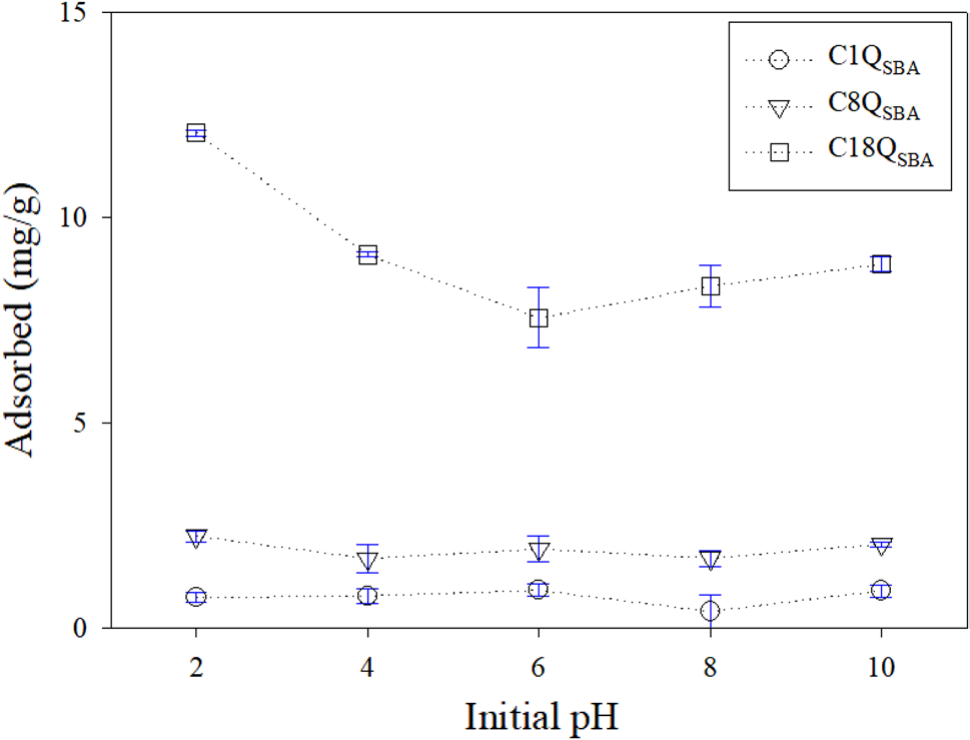
Effect of pH on CBZ adsorption by Q_SBA_.

In a previous study, the adsorption of DCF onto C8Q_SBA_ was reduced after an increase in the initial pH from 5 to 12. This suggests that DCF adsorption onto C8Q_SBA_ involves not only hydrophobic interactions but also an anion exchange with the N^+^ moiety of the quaternary ammonium group. Unlike CBZ, DCF dissociates into negatively charged molecules at pH > 4.15 (pK_a_ = 4.15)^35^. Consequently, the pH controls both the hydrophobic and hydrophilic interactions and determines the adsorption efficiency of DCF by Q_SBA_. Figure 4 shows the effect of the initial pH on CBZ removal by Q_SBA_. Despite the variations in the initial pH, stable adsorption capacities were observed (0.41–0.92, 1.70–2.24, and 7.56–9.10 mg/g for C1Q_SBA_, C8Q_SBA_, and C18Q_SBA_, respectively); the exception was when the pH was 2 and C18Q_SBA_ was used. Thus, the pH had a limited effect on the interactions between CBZ and Q_SBA_. However, C18Q_SBA_ showed an improved adsorption capacity of 12.06 ± 0.07 mg/g at pH 2. The pH of the CBZ solution was adjusted using HCl and NaOH. Thus, it was expected that an extremely low pH would improve the hydrophobic interactions at high concentrations of H^+^ and Cl^-^. The H^+^ and Cl^−^ concentrations at pH 2 were 10 mM. It is known that when ions and proteins are present in high concentrations, they compete to interact with the water molecules, and unreacted proteins are precipitated by the hydrophobic interactions^57^. Similarly, Bautista-Toledo et al.^58^ explained that the enhancement in the adsorption of sodium dodecylbenzenesulfonate (SDBS) on activated carbon with increasing ionic strength was owing to the decreased solvation of SDBS because of the high ionic strength, which increased the hydrophobic-interaction-based adsorption. Likewise, the high concentrations of H^+^ and Cl^−^ may have enhanced the hydrophobic interactions between C18Q_SBA_ and CBZ in the present study. Similarly, the Na^+^ and OH^−^ ions probably also aided the enhancement in the hydrophobic interactions when present in a high concentration at pH 10. However, the concentrations of Na^+^ and OH^−^ were 0.1 mM at pH 10 and only 1/100th of those of H^+^ and Cl^−^ at pH 2. The effect of the ion strength on the hydrophobic interactions between CBZ and Q_SBA_ was verified using NaCl, as described in the next section.

### Effect of ionic strength

**Figure 5:**
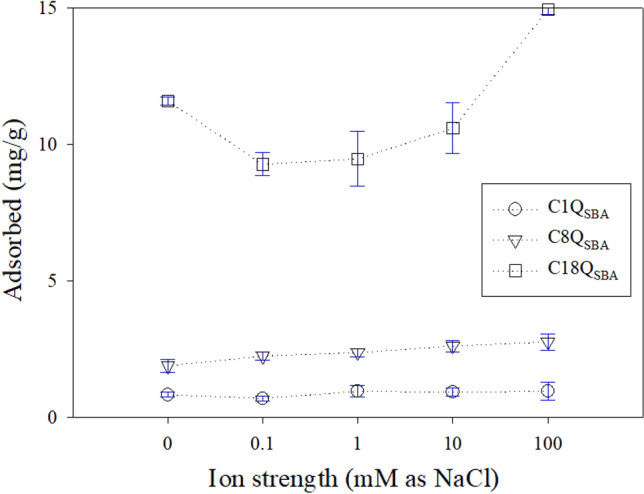
Effect of ionic strength on CBZ adsorption by Q_SBA_.

Figure 5 shows the effect of the ionic strength on the adsorption of CBZ by Q_SBA_. The adsorption capacity increased with increasing ionic strength. The amounts of CBZ adsorbed were 0.70 ± 0.09, 2.25 ± 0.14, and 9.27 ± 0.42 mg/g for C1Q_SBA_, C8Q_SBA_, and C18Q_SBA_ when 0.1 mM NaCl was used. In contrast, the adsorption capacities increased to 0.96 ± 0.33, 2.77 ± 0.29, and 14.94 ± 0.17 mg/g for C1Q_SBA_, C8Q_SBA_, and C18Q_SBA_, respectively, in the case of 100 mM NaCl. These values are 1.38, 1.23, and 1.61 times higher, respectively, than the corresponding ones for 0.1 mM NaCl. The high salt concentration enhanced the hydrophobic interactions and shielded the electrostatic attraction between CBZ and the N^+^ moiety of Q_SBA_^32,59^. Interestingly, only in the case of C18Q_SBA_ did the adsorption capacity exhibit high sensitivity to the ionic strength, with C18Q_SBA_ showing an enhanced adsorption capacity of 10.60 ± 0.92 mg/g when 10 mM NaCl was used. The ionic concentrations in this case were similar to those at pH 2. Thus, it was confirmed that the hydrophobic interactions are predominantly controlled by the ionic strength and not the pH. The reason for the highest sensitivity of C18Q_SBA_ was not clear. However, we believe that its longer alkyl chain exhibits stronger hydrophobic interactions in saline conditions. This means that the alkyl chain length can be increased to improve the efficiency of CBZ removal from wastewater samples with high ionic strength. The critical ionic strength of 100 mM NaCl (≒ 12–20 mS/cm^60,61^) is higher than that of surface water (0.9 mS/cm), sanitary sewage (1.5–3.0 mS/cm), and treated water (0.22–0.37 mS/cm) but similar to that of industrial wastewater (35–70 mS/cm) and sea water (30 mS/cm)^62–64^. This characteristic should allow for a high CBZ adsorption capacity in strongly ionic wastewaters. However, additional investigations are required to confirm the applicability of Q_SBA_ for use with wastewaters with high ionic strength.

## Conclusions

Batch experiments were performed to investigate the primary mechanism of CBZ adsorption on Q_SBA_ with different alkyl chain lengths. The efficiency of CBZ absorption by Q_SBA_ increased with increases in the reaction time and alkyl chain length of Q_SBA_. Based on the Langmuir isotherm model, the maximum sorption capacities for C1Q_SBA_, C8Q_SBA_, and C18Q_SBA_ were determined to be 3.14, 6.56, and 24.5 mg/g, respectively. Regarding the effect of the initial pH, the adsorption capacity was mostly stable within the pH range of 4–10; the exception was a pH of 2. Because the pk_a_ value of CBZ is 13.9, undissociated CBZ does not interact with the N^+^ ions of Q_SBA_. Consequently, it was assumed that hydrophobic interactions would be dominant in the pH range investigated in this study. The increase in CBZ adsorption at higher ionic strengths (similar to those of actual wastewater) is also attributable to the hydrophobic interactions between the alkyl chains of Q_SBA_ and CBZ. Thus, by controlling the alkyl chain length of Q_SBA_, we were able to elucidate the CBZ adsorption mechanism in detail. Moreover, by comparing the adsorption characteristics of various PPCPs by adjusting the alkyl chain length of Q_SBA_, it should not only be possible to determine the adsorption mechanism but also use the appropriate adsorbent for PPCPs based on their characteristics such as their hydrophobicity.

## Supplementary Information

Supplementary Information.

## Data Availability

The datasets used and/or analysed during the current study available from the corresponding author on reasonable request.
