# Supplementary material for: Alkyl chain length of quaternized SBA-15 and solution conditions determine hydrophobic and electrostatic interactions for carbamazepine adsorption
Source: Sci Rep. 2023 Mar 30;13:5170. doi: 10.1038/s41598-023-32108-3 (PMC10063578; doi:10.1038/s41598-023-32108-3)
Supplement: Supplementary file 1 — Supplementary Information. [file 41598_2023_32108_MOESM1_ESM.docx]

**Alkyl chain length of quaternized SBA-15 and solution conditions determine hydrophobic and electrostatic interactions for carbamazepine adsorption**

Jin-Kyu Kang ^a^, Hyebin Lee ^b^, Song-Bae Kim^c^, Hyokwan Bae ^d,e,*^

^a^Institute for Environment and Energy, Pusan National University, 2 Busandaehak-ro 63beon-gil, Geumjeong-gu, Busan 46241, Republic of Korea

^b^Department of Civil and Environmental Engineering, 2 Busandaehak-ro 63beon-gil, Geumjeong-gu, Pusan National University, Busan 46241, Republic of Korea

^c^Environmental Functional Materials and Water Treatment Laboratory, Department of Rural Systems Engineering, Seoul National University, 1 Kwanak-ro, Kwanak-gu, Seoul 08826, Republic of Korea

^d^Department of Urban and Environmental Engineering, Ulsan National Institute of Science and Technology (UNIST), 50 UNIST-gil, Eonyang-eup, Ulju-gun, Ulsan 44919, Republic of Korea

^e^Graduate School of Carbon Neutrality, Ulsan National Institute of Science and Technology (UNIST), 50 UNIST-gil, Eonyang-eup, Ulju-gun, Ulsan 44919, Republic of Korea

*Corresponding author**:** Hyokwan Bae, PhD

Department of Urban and Environmental Engineering, Ulsan National Institute of Science and Technology (UNIST), 50 UNIST-gil, Eonyang-eup, Ulju-gun, Ulsan 44919, Republic of Korea E-mail: hyokwan.bae@unist.ac.kr

Tel: +82-52-217-2801

Fax: +82-52-217-2849


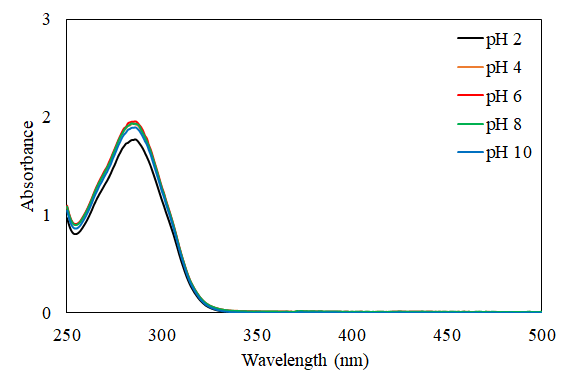


**Figure S1. Absorbance spectra of CBZ (40 mg/L) from pH 2 to 10**


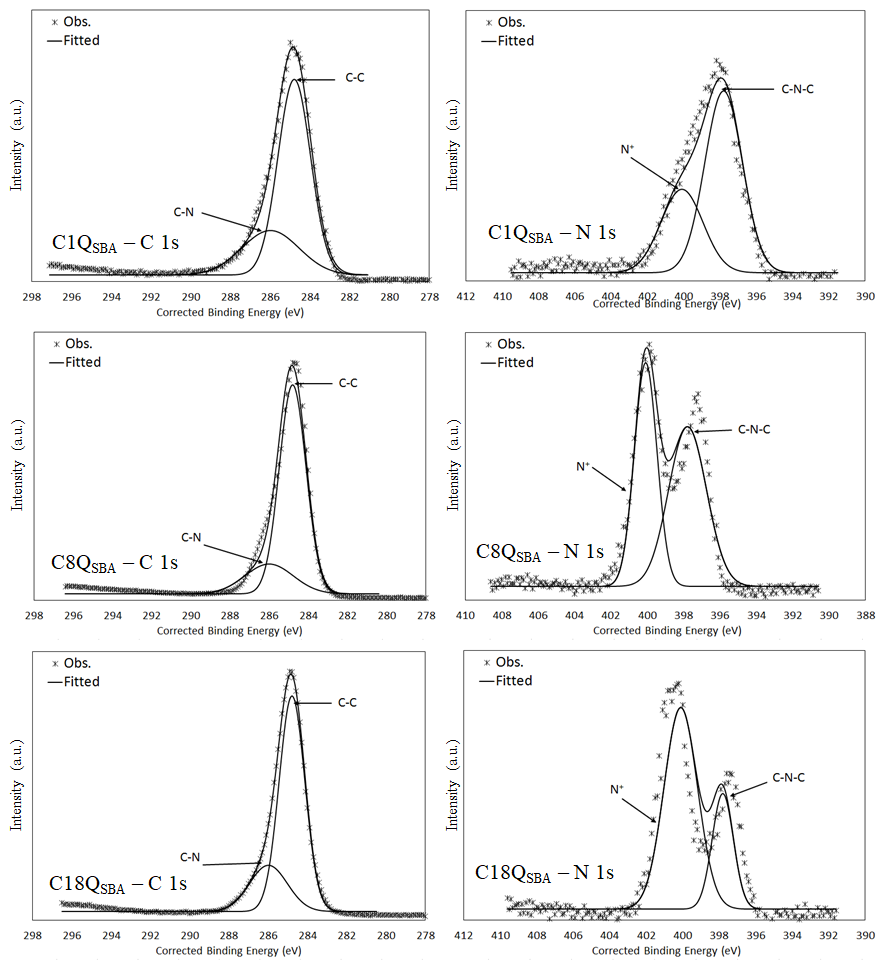


Figure S2. C1s and N1s XPS spectra of C1Q_SBA_, C8Q_SBA_, and C18Q_SBA_. Figure was modified from Kang and Kim^35^_._


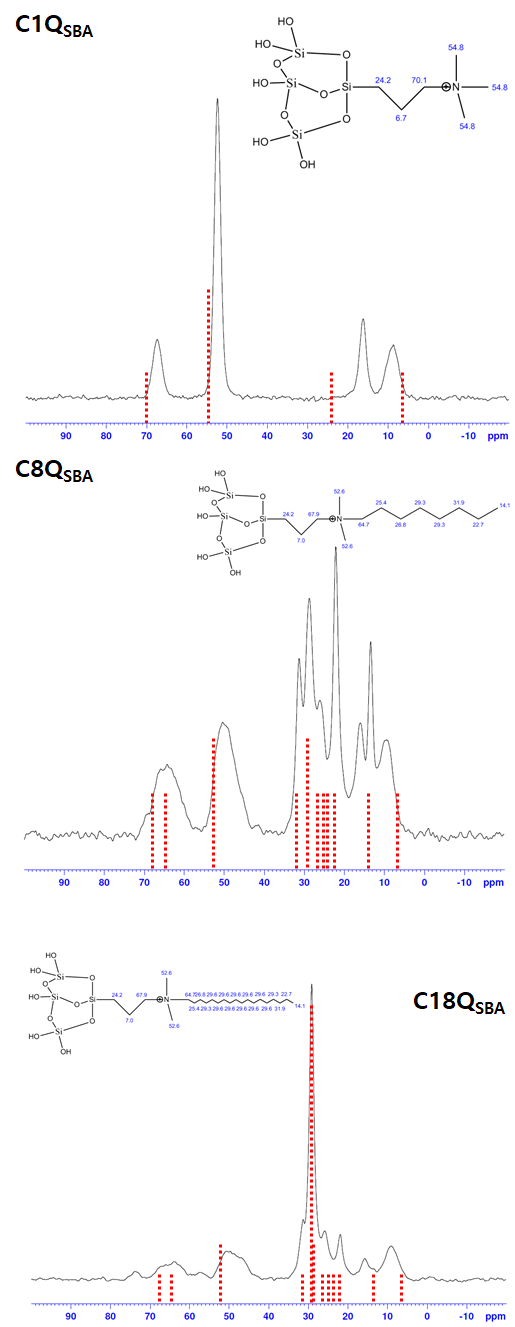


Figure S3. Measured solid state ^13^C NMR spectra (black line) and estimated ^13^C NMR spectra (red dotted line) of Q_SBAs_ (estimated using ChemDraw 16, PerkinElmer Informatics, USA). Figure was modified from Kang and Kim^35^_._


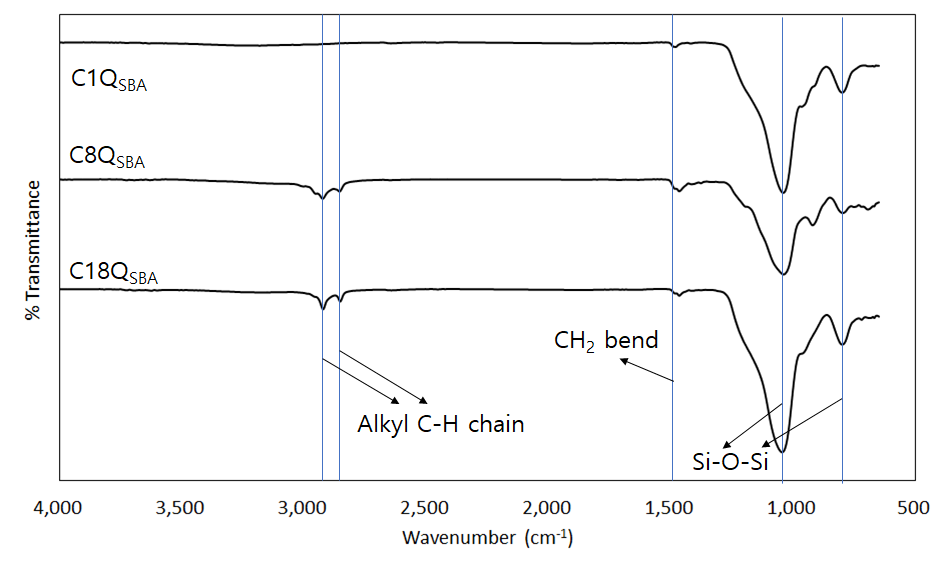


Figure S4. FT-IR spectra of C1Q_SBA_, C8Q_SBA_, and C18Q_SBA_. Figure was modified from Kang and Kim^35^_._


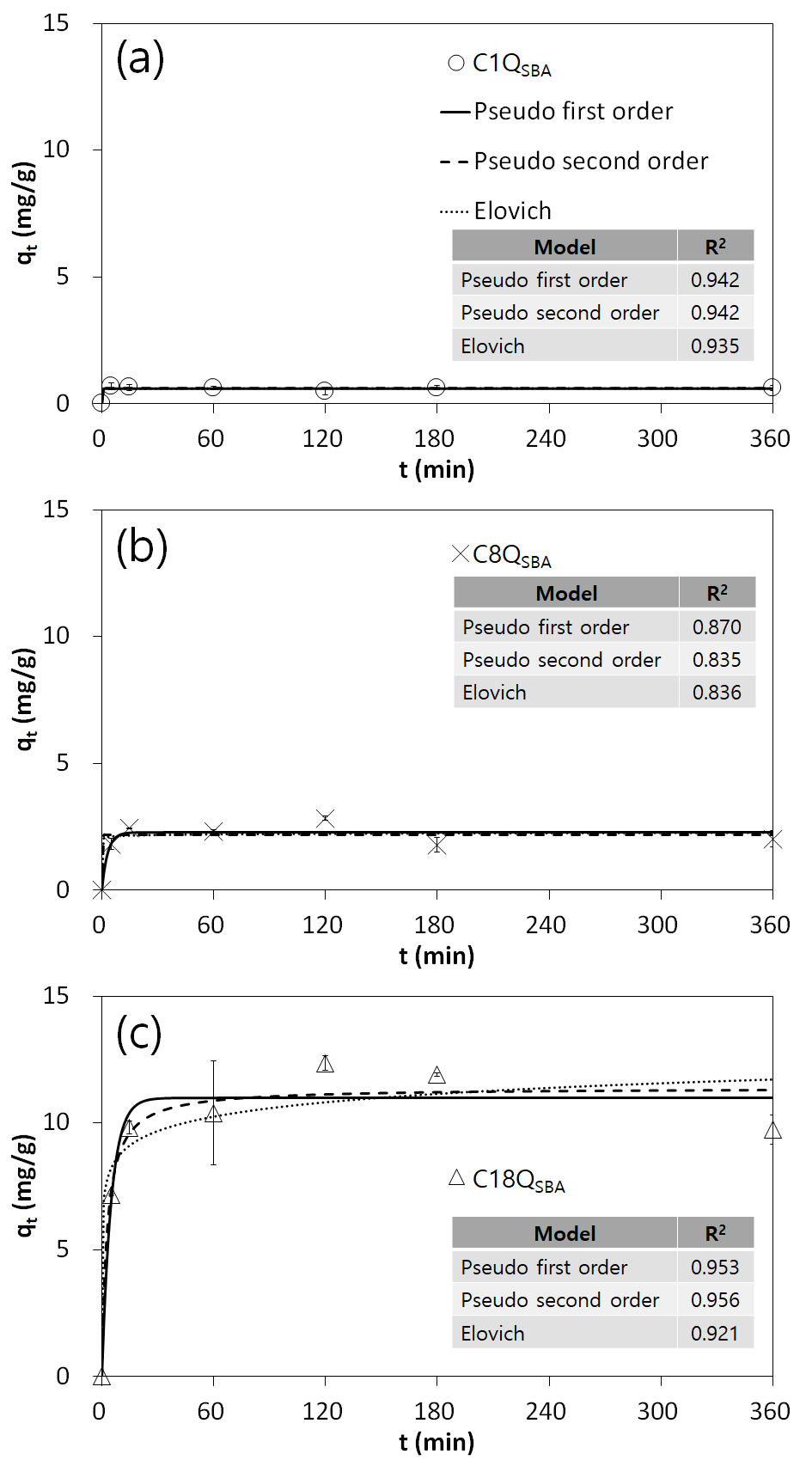


**Figure S5. Kinetic model fitted results for CBZ adsorption by Q_SBA_; (a) C1Q_SBA_, (b) C8Q_SBA_, and (c) C18Q_SBA_**


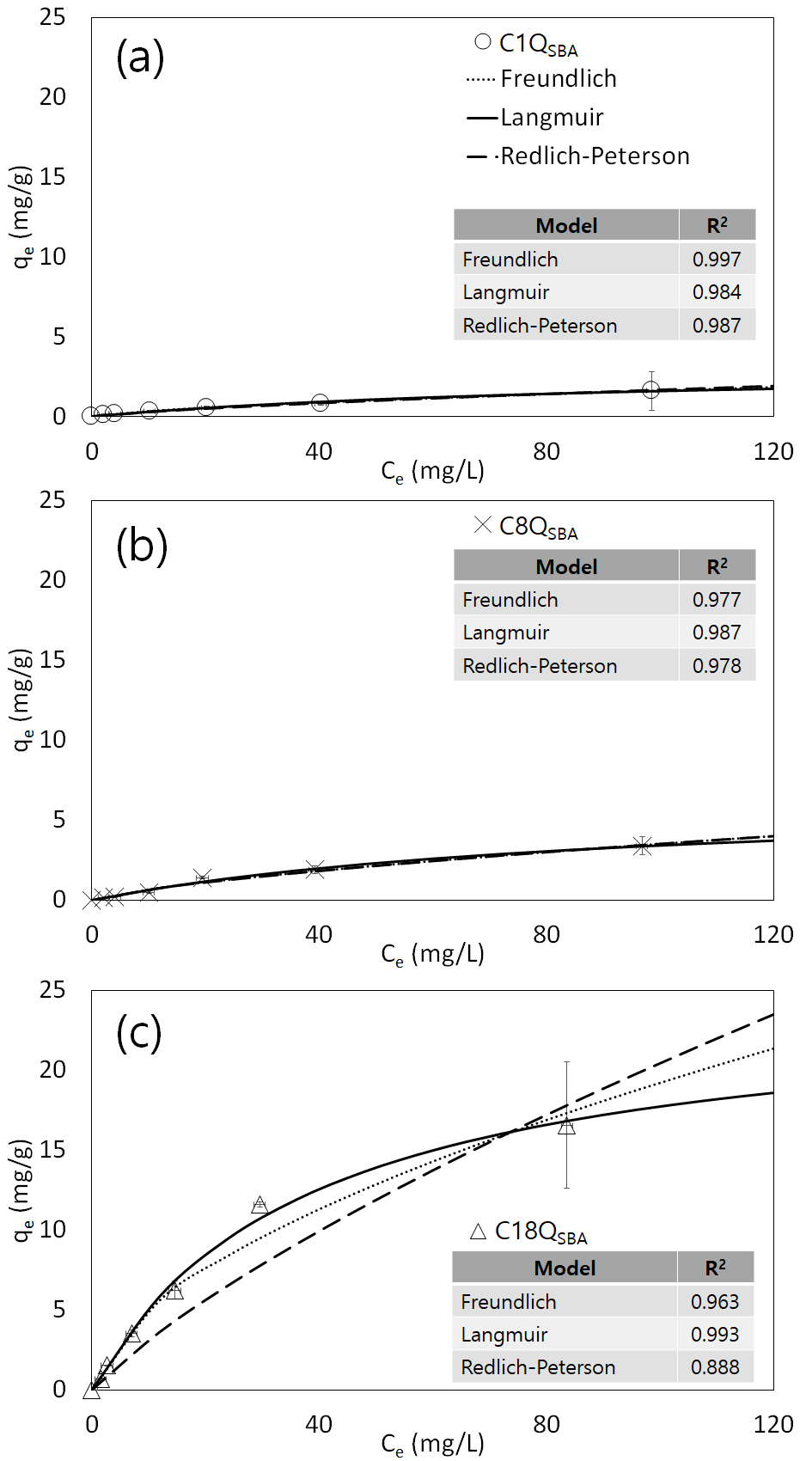


**Figure S6. Isotherm model fitted results for CBZ adsorption by Q-SBA-15; (a) C1Q-SBA-15, (b) C8Q-SBA-15, and (c) C18Q-SBA-15**

Table S1. The detailed experimental conditions for the batch experiments

| Batch experiments | Experimental conditions | | | | |
| --- | --- | --- | --- | --- | --- |
|  | Dose (g/L) | Initial concentrations (mg/L as CBZ) | Reaction time (min) | Initial pH (adjusted with 0.1 M HCl and 0.1 M NaOH) | Ion strength (mM as NaCl) |
| Reaction time | 1 | 40 | 5, 15, 60, 120, 180, and 360 | Not adjusted | 0 |
| CBZ concentration | 1 | 2, 5, 10, 20, 50, 100 | 360 | Not adjusted | 0 |
| pH | 1 | 40 | 360 | 2, 4, 6, 8, and 10 | 0 |
| Ion strength | 1 | 40 | 360 | Not adjusted | 0, 0.1, 1, 10, 100 |

Table S2. The detailed experimental conditions for the batch experiments

| Batch experiments (Applied models) | Models | Equations (Non-linear form) |
| --- | --- | --- |
| Reaction time  (Kinetic models) | Pseudo first-order | $q_{t}=q_{e}[1-exp(-k_{1}t)]$ |
|  | Pseudo second-order | $q_{t}=\frac{k_{2}{q_{e}}^{2}t}{1+k_{2}q_{e}t}$ |
|  | Elovich | $q_{t}=\frac{1}{\beta}\ln\left( \alpha\cdot\beta\right)+\frac{1}{\beta}ln(t)$ |
| Initial concentration  (Isotherm models) | Freundlich | $q_{e}=K_{F}{C_{e}}^{1/n}$ |
|  | Langmuir | $q_{e}=\frac{Q_{m}K_{L}C_{e}}{1+K_{L}C_{e}}$ |
|  | Redlich-Peterson | $q_{e}=\frac{K_{R}C_{e}}{1+a_{R}C_{e}^{g}}$ |

where *k_1_* is the pseudo first order rate constant (1/min), *k_2_* is the pseudo second order rate constant (g/mg/min), *q_e_* is the amount of CBZ adsorbed at equilibrium (mg/g), *q_t_* is the amount of CBZ adsorbed at time *t* (mg/g)*, α* is the initial removal rate constant (mg/g/min), and *β* is the Elovich constant (g/mg). where *K_F_* is the Freundlich constant, *C_e_* is the equilibrium concentration in the aqueous solution, *1/n* is the Freundlich constant, *Q_m_* is the maximum adsorption capacity*, K_L_* is the Langmuir constant, *K_R_* is the Redlich-Peterson removal constant, *a_R_* is the Redlich-Peterson constant and *g* is the Redlich-Peterson constant.

Table S3. Comparison of sorption capacity of carbamazepine by various adsorbents

| PPCPs | Adsorbents | Q_max_ (mg/g) | Reference |
| --- | --- | --- | --- |
| Carbamazepine | SBA-15 | 0.200 | Suriyanon, et al. ^1^ |
|  | Mercapto-functionalized HMS derivatives | 0.217 | Suriyanon, et al. ^1^ |
|  | 4-vinylbenzoic acid based molecular imprinted adsorbent | 28.4 | He, et al. ^2^ |
|  | Metal organic framework (UiO-66(Zr)) | 37.2 | Chen, et al. ^3^ |
|  | Carbon dot-modified magnetic carbon nanotubes | 104.17 | Deng, et al. ^4^ |
|  | Metal organic framework (Basolite A100) | 250.4 | Jun, et al. ^5^ |
|  | C18Q_SBA_ | 24.5 | This study |

**Reference**

1 Suriyanon, N., Punyapalakul, P. & Ngamcharussrivichai, C. Mechanistic study of diclofenac and carbamazepine adsorption on functionalized silica-based porous materials. *Chem. Eng. J.* **214**, 208-218, doi:https://doi.org/10.1016/j.cej.2012.10.052 (2013).

2 He, Q. *et al.* Removal of the environmental pollutant carbamazepine using molecular imprinted adsorbents: Molecular simulation, adsorption properties, and mechanisms. *Water Res.* **168**, 115164, doi:https://doi.org/10.1016/j.watres.2019.115164 (2020).

3 Chen, C. *et al.* Adsorption Behaviors of Organic Micropollutants on Zirconium Metal–Organic Framework UiO-66: Analysis of Surface Interactions. *ACS Applied Materials & Interfaces* **9**, 41043-41054, doi:10.1021/acsami.7b13443 (2017).

4 Deng, Y., Ok, Y. S., Mohan, D., Pittman, C. U. & Dou, X. Carbamazepine removal from water by carbon dot-modified magnetic carbon nanotubes. *Environ. Res.* **169**, 434-444, doi:https://doi.org/10.1016/j.envres.2018.11.035 (2019).

5 Jun, B.-M., Heo, J., Park, C. M. & Yoon, Y. Comprehensive evaluation of the removal mechanism of carbamazepine and ibuprofen by metal organic framework. *Chemosphere* **235**, 527-537, doi:https://doi.org/10.1016/j.chemosphere.2019.06.208 (2019).
